# Alternatives to direct emergency department conveyance of ambulance patients: a scoping review of the evidence

**DOI:** 10.1186/s13049-020-00821-x

**Published:** 2021-01-06

**Authors:** Joanna M. Blodgett, Duncan J. Robertson, Elspeth Pennington, David Ratcliffe, Kenneth Rockwood

**Affiliations:** 1 MRC Unit for Lifelong Health and Ageing, UCL, 1-19 Torrington Place, London, WC1E 7HB UK; 2 North West Ambulance Service, NHS Trust, Bolton, UK; 3 Division of Geriatric Medicine, Department of Medicine, Dalhousie University, Halifax, Canada; 4 Welsh Ambulance Services NHS Trust, Denbighshire, UK; 5 Greater Manchester Health and Social Care Partnership, Manchester, UK

**Keywords:** Ambulance, Alternative care routes, Non-emergency medical care, Pre-hospital emergency care, Scoping review, Referrals

## Abstract

**Background:**

The role of ambulance services is shifting, due in part to more intermediate, non-urgent patients who do not require direct emergency department conveyance, yet who cannot be safely left at home alone. Evidence surrounding the safety, effectiveness and efficiency of alternate care routes is not well known.

**Methods:**

This scoping review sought to identify all studies that examined alternate routes of care for the non-urgent “intermediate” patient, as triaged on scene. Search terms for the sample (ambulances, paramedics, etc.) and intervention (e.g. referrals, alternate care route, non-conveyance) were combined. Articles were systematically searched using four databases and grey literature sources (February 2020). Independent researchers screened title-abstract and full text stages.

**Results:**

Of 16,037 records, 41 examined alternate routes of care after triage by the on-scene paramedic. Eighteen articles considered quantitative patient data, 12 studies provided qualitative perspectives while 11 were consensus or opinion-based articles. The benefits of alternative schemes are well-recognised by patients, paramedics and stakeholders and there is supporting evidence for a positive impact on patient-centered care and operational efficiency. Challenges to successful use of schemes included: patient safety resulting from incorrect triage decisions, inadequate training, lack of formal partnerships between ambulance and supporting services, and insufficient evidence to support safe implementation or continued use. Studies often inaccurately defined success using proxies for patient safety (e.g. decision comparisons, rates of secondary contact). Finally, patients expressed willingness for such schemes but their preference must be better understood.

**Conclusions:**

This broad summary offers initial support for alternate routes of care for intermediate, non-urgent patients. Even so, most studies lacked methodologically rigorous evidence and failed to evaluate safe patient outcomes. Some remedies appear to be available such as formal triage pathways, targeted training and organisational support, however there is an urgent need for more research and dissemination in this area.

## Introduction

Ambulance trusts continue to experience annual increases in the number of emergency calls. In 2000, the UK Ambulance services responded to 4.41 million calls per year [1]. By 2018–2019, call frequency had more than tripled, reaching a record high of 13.8 million calls [2–5]. However, a large proportion of these emergency calls do not require emergency department (ED) attendance [6, 7]. While some patients can be safely left at home, many others are conveyed to hospital for non-urgent care and contribute to ED overcrowding [8]. Limitations in ED capacity can lead to long delays in corridor wait time, reduced availability of ambulances for subsequent emergencies and rising health care costs [9–11].

While 999 dispatch processes aim to triage patients at the point of call, it can be difficult to ascertain what a patient needs until there is a healthcare practitioner on scene. When on scene, paramedics often triage the patient to determine necessity of direct ED conveyance or if it is safe for the patient to be left at home [12]. Even so, this leaves an important group of “intermediate” patients, who now are the driving force behind increased ED attendance. There is no clear definition of such patients, although the UK Paramedic Pathfinder triage system defines these patients as amber, while the Swedish Rapid Emergency Triage and Treatment System (RETTS) triages them as yellow or green. Based on these objective definitions, we define “intermediate” patients as those with non-urgent medical illnesses or injuries who may not require conveyance to the ED, yet cannot be safely left at home without medical support [13]. Due to a lack of access to alternative routes of care, these intermediate patients are often unnecessarily conveyed to ED [14]. Non-urgent patients account for up to 50% of all ED attendances [15, 16], suggesting that there is a need for ambulance services to target alternative routes of care for such patients.

At both the national and international level, there is no consensus on alternative routes of care to direct ED conveyance. With a distinct lack of empirical evidence, a collaborative effort across ambulance trusts is required to identify potential benefits or consequences for the individual and the healthcare system as a whole [17]. Older systematic reviews document few alternative care pathways and insufficient evidence to deduce whether they are safe [18]. Jensen et al. [19] recently catalogued outcomes of alternative emergency medical services (EMS) dispatch and transportation programs but, to our knowledge, there is no consensus understanding of the protocols of such schemes nor of the supporting evidence. Interventions that allow paramedics to appropriately direct individuals to alternative care pathways can ensure patient safety, improve ambulance and ED efficiency, whilst also providing substantial savings to the healthcare system [20] .

As different ambulance trusts in varying countries begin to explore alternative routes of care for these intermediate patients, synthesis of current programs, services and protocols is crucial. To avoid ambulance services working in isolation, it is important to recognize what different services have implemented, and whether or not these services are safe and beneficial to the patient and the health care system. Given the lack of evidence in this area, this scoping review had the following objectives:

1. to identify all studies that examined alternatives to direct ED conveyance for patients triaged by the on-scene emergency medical clinician;
2. to describe all alternative schemes and study outcomes in the identified studies and;
3. to assess the quality of the evidence provided.

Due to our prior research in this field [16], we hypothesised that there would be strong heterogeneity of alternative non-ED schemes across various emergency medical services, a low quality of evidence and limited evidence that adequately assessed patient outcomes.

## Methods

Scoping reviews are used to map the key concepts that constitute the groundwork of a specific area as well as the main source and type of evidence available [21]. Here, we aim to attain a representative and near-comprehensive sample of the evidence in a pre-defined topic area and to describe the quality of the evidence base available in terms of study design and questions addressed by the identified studies [22]. The methodology of this review follows the Preferred Reporting Items for Systematic Reviews and Meta-Analyses extension for Scoping Reviews (PRISMA-ScR) statement [23].

### Search strategy

We used initial pilot searching in two databases to aid in defining effective search terms (JB, DR, additional librarian support). This iterative process provided confidence that the search strategy was adequately capturing all relevant studies. The resulting search strategy remained purposely broad and thus the numbers of studies captured by the search was expected to be large. The initial search strategy was conducted in April 2016 but was updated in April 2017 and February 2020. We searched PubMed, CINAHL, Web of Science and ProQuest Health & Medicine databases. Two separate arms were combined using the Boolean operator “AND” to search for the *“who”* (paramedic or ambulance) and the *“what”* (referral or non-conveyance). The full search strategy can be found in Appendix; it was adapted as necessary for each additional database. To ensure all relevant studies were adequately captured, no exclusion filters were applied for language, year (e.g. inception to February 2020) or article type. We also searched grey literature on the following sites: NHS Evidence, CORE, BL.UK, OpenGrey, and HMIC. Additional articles were found by a snowball search of the reference lists of relevant systematic reviews and of articles that met the inclusion criteria.

### Study selection

In the first stage of screening, two researchers independently screened the title and abstracts of each article for relevancy (JB, DR, EP). The second stage of screening involved retrieval and evaluation of the full text to identify if it met the criteria. Again, two of these three researchers independently screened the full text of all potential studies. Reviewers discussed any discrepancies and where necessary, a third reviewer made the final decision. Reason for exclusion in the full text stage was noted.

Inclusion criteria purposely remained broad to capture relevant studies. Articles were included if they considered emergency service callers (e.g. 999, 911, etc.) who were triaged by the on-scene clinician as non-urgent “intermediate” patients and if there was an indication of an alternate route of care to conveyance to ED. “Intermediate” patients were those with non-urgent medical illnesses or injuries who did not require conveyance to the ED, yet could not be safely left at home without medical support [13]. Emergency department is defined as a medical facility responsible for treatment of patients who arrive at the hospital and require immediate medical care. Undifferentiated “intermediate” patients who did not fit within an existing evidence-based pathway were included, while samples of specific clinical patients with pre-existing evidence-based pathways were excluded (e.g. falls, resolved hypoglycaemia, psychiatric and resolved epilepsy pathways). Commentaries, protocols and policy statements were eligible for inclusion, while literature reviews, conference abstract, non-English and non-peer reviewed articles were excluded from final article selection. Due to the expected heterogeneity of studies, there were no limitations on study outcomes. The following software were used in study selection and screening: Mendeley, Microsoft Excel and Rayyan.

### Data extraction

JB independently extracted data from all studies using a standardised form that was agreed upon and piloted by the research team (JB, DR, EP). This included data on country, study type, sample size, description of alternate route of care, triage protocol, study outcomes and study findings. A meta-analysis was not possible due to expected heterogeneity of included studies, and a narrative synthesis was conducted instead. The heterogeneity in design, methods and outcomes of studies also facilitated three groups for synthesis: 1) quantitative patient-focused, evidence-based studies; 2) qualitative, evidence-based studies; and 3) consensus-based articles.

In line with the second objective, two authors (JB, EP) independently appraised the quality of evidence following a modified 7-level rating system for the hierarchy of evidence [24]. This tool provides a hierarchy of the likely best evidence (e.g. Levels 1 to 7) and is specifically designed to aid clinicians (and patients) with a rapid appraisal to avoid the need to resort to original sources. Differences in levels of evidence between authors were discussed and agreed upon in a consultative process.

## Results

**Fig. 1:**
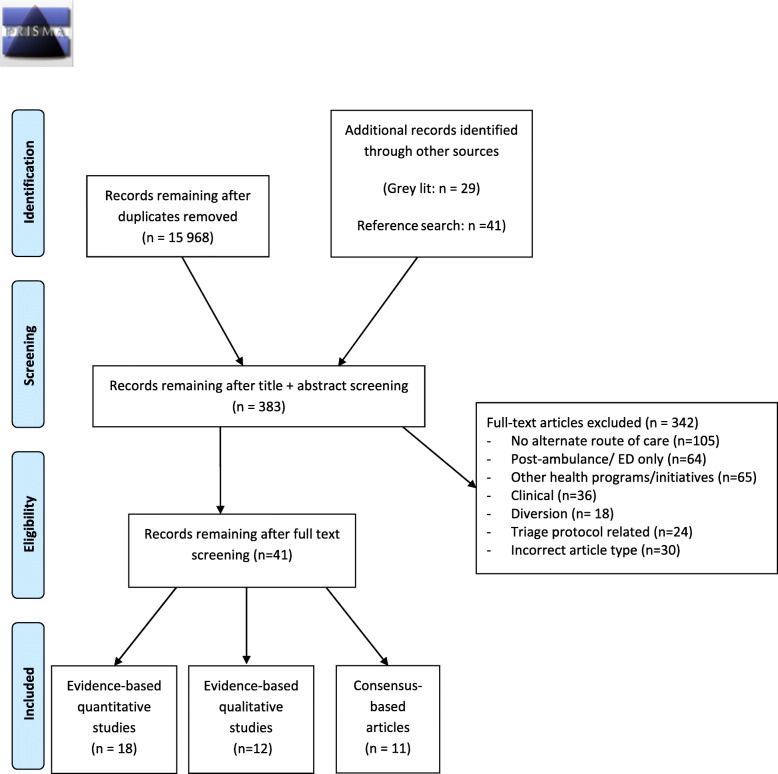
PRISMA flow chart indicating identification, screening stages, and final inclusion of studies

The search revealed 15,968 single records from databases and 69 from grey literature and reference list screening. After title and abstract screening, 383 records remained for the full text screening stage. Most articles were excluded as they did not mention an alternate route of care (*n* = 105), focused on ED related issues (*n* = 64) or other public health initiatives (*n* = 65) including pre-ambulance care, walk-in primary care, community paramedicine, air ambulance or other emergency professions (firefighting, police). Other exclusions were for specific clinical problems (*n* = 36), diversion to other emergency hospital facilities (*n* = 18), triage related (*n* = 24) or wrong article type (*n* = 30). Of the 41 included studies, there were 18 quantitative evidence-based studies, 12 qualitative evidence-based studies and 11 consensus-based articles including commentaries, protocols and policies. A PRISMA flow-diagram outlining study screening and selection is shown in Fig. 1.

Levels of evidence of all 41 studies were graded from 1 to 7, as described above [24]. Of the 18 quantitative studies, two were graded as a Level 2 due to their random allocation of intervention and control groups, 11 studies were graded as Level 4, four studies as Level 3 and a single descriptive study was graded as a Level 6. All 12 qualitative studies were graded as a 6; while they demonstrated perceived support for such schemes from individuals involved, they did not contribute evidence to understanding if the scheme is safe and effective. Finally, all consensus-based papers were graded as a 7 (i.e. expert opinion), given that they did not contribute any evidence to the field.

### Quantitative, evidence based studies

Eighteen studies with patient-focused, quantitative evidence are outlined in Table 1. Seven studies were based in the UK, four in both the USA and Sweden and one in each of Canada, Australia and the Netherlands. Only five studies compared an intervention group (e.g. option to refer to alternate care route) and a control group (e.g. normal practice), with two of these studies randomising into either arm [37, 41]. Retrospective cohorts, including data audits, were the most common study design (*n* = 8), followed by prospective cohorts (*n* = 7) and randomised control trials (RCTs) (*n* = 2). One study used a comprehensive mixed-methods approach of interviews, qualitative telephone data and linked retrospective data [32]. Most studies (*n* = 14) outlined a triage protocol to guide the ambulance clinician’s decision making; this included six studies with triage tools that led directly to alternate care route outcomes, five studies with a series of protocols for specific incidents and three studies that allowed subjective referral of patients triaged as low acuity using traditional triage tools. Studies commonly detailed multiple alternative care routes that could be accessed through either ambulance transport or referral. Referrals to primary care, including general practitioners (GPs) or nurses, were most frequent (*n* = 16), although options such as urgent care centres (*n* = 6), psychiatric or social teams (*n* = 4) and minor injury units (*n* = 2) were also common.

**Table 1:** Summary of quantitative, evidence based studies (*n* = 18)

First author year (ref)	Country & sample size	Study design	Study aim	Triage protocol to determine eligibility for alternate route of care	Description of alternate route of care	Findings	Concluding evidence (and level of support)	Level of Evidence ^**+**^
Blodgett 2020 [25]	UK *n* = 5283	Pilot data linkage of retrospective patient data	To determine feasibility linking data to assess differences in patients conveyed directly to ED and those referred to a GP referral scheme	Paramedic Pathfinder (protocol tool outlining alternate routes of care)	Referral to partner GP providers	– Patients were more likely to be referred to GP if they were: i. women ii. older iii. Lower priority at dispatch. – 22% of referred patients presented and 13% were admitted to ED w/i 30 days. – There was no difference in hospital outcomes between GP-referred and directly conveyed groups.	**Positive support** – GP referral scheme provides a safe alternative path of care and does not increase risk of poor outcomes. – Recommendation for a large-scale study to provide evidence-based recommendations for changes to EMS care pathways.	3
Ebben 2019 [26]	Netherlands *n* = 426	Retrospective observational study	To describe characteristics of non-conveyance ambulance incidents	National protocol for EMS decisions	Referral to GP/medical specialist	– 31.1% of patients in a 12-month period were not conveyed. – 36.6% of non-conveyed patients were referred to GP and 6.1% to medical specialist.	**Inconclusive support** – A significant number of ambulance visits end in non-conveyance. – Note, differences between those conveyed, referred and left at home were not examined.	6
Krumperman 2005 [27]	USA *n* = 2143	Retrospective cohort	To compare patient satisfaction and referral adherence in two systems: i. “evaluate, treat and refer” ii. “telephone triage and referral”	No description of triage process	Referral to: i. primary care provider ii. urgent care centre	– Patients evaluated and referred by paramedic were less likely to follow instructions than those referred by telephone [odds ratio: 0.31 (0.14–0.69)]. – Patients were highly satisfied with the alternate route of care.	**Positive support** – Systems that use both pre-ambulance telephone triage and on-scene referral pathways can help avoid unnecessary ED visits.	4
Larsson 2017 [28]	Sweden *n* = 394	Prospective cohort study compared with a matched retrospective control group	To examine pre-hospital assessment of non-urgent patients, and investigate outcomes of different levels of care	Rapid Emergency Triage and Treatment System (RETTS)	- Consulted with GP to decide alternate route: i. primary home healthcare supervision ii. transportation to primary healthcare unit	– Intervention group resulted in: i. decreased ED conveyance (17.4%; 53.1%) ii. no difference in transport to primary care unit (8.7%; 10.4%) iii. Reduced on-scene ambulance time (87 min; 94 min) iv. decreased hospital admissions (11.4%; 25.6%) v. no additional secondary transport w/i 48 h (7.9%; 8.0%).	**Positive support** – Collaboration between ambulance nurses and GPs can improve appropriate level of care for non-urgent patients and safely decrease unnecessary ED conveyance.	3
Magnusson 2016 [29]	Sweden *n* = 529	Retrospective observational study	To describe characteristics, assessments, and routes of care of low priority patients (as assessed by dispatcher)	RETTS	Referral to: i. primary care appointment ii. community nurse iii. Mobile psychiatric or social care team.	– Compared to ED-conveyed patients, patients who were referred or given self-care advice: i. were younger ii. required a shorter job time. – Of those referred or left at home, 19% (visited ED within 72 h; half of these were admitted and a further half of those admitted required intervention/treatment).	**Mixed support** – Single-responder nurse can safely triage to the appropriate level of care, providing more effective use of emergency services. – Note that the study did not solely consider a group of referred patients (e.g. combined with self-care patients) so conclusions specific to referrals cannot be made.	4
Magnusson 2020 [30]	Sweden *n* = 6712	Prospective cohort	To assess patient characteristics and evaluate appropriateness of: i. initial triage and; ii. non-transport decisions	RETTS- Adults	Referral to: i. primary care; ii. social or home care	– Compared to ED-conveyed patients, non-conveyed patients were more likely: i. to be younger ii. to be women iii. have no medical history iv. have better vital signs v. to have been lower priority at initial dispatch. – 10% of non-conveyed patients were admitted to ED within 72 h (1% considered time-critical).	**Mixed support** – Defining patient characteristics that may help initial assessment. – Improved assessment tools, appropriate use of full triage and better education is necessary.	4
Newton 2013 [31]	UK *n* = 481	Prospective cohort	To evaluate if paramedics can safely use Paramedic Pathfinder to direct patients into alternate routes of care	Paramedic Pathfinder (protocol tool outlining alternate routes of care)	– Two alternate routes: i. community care pathway (referral to ambulance GP) ii. transport to urgent care centre	– There was high agreement in decision-making between expert senior medical practitioners and ambulance clinicians. – Sensitivity (95%) and specificity (58%) of the tool were sufficient.	**Positive support** – Ambulance clinicians can successfully use Paramedic Pathfinder to identify patients that do not require ED care. – The potential benefits of using the tool fully depend on provision of suitable community alternatives.	4
O’Cathain 2018 [32]	UK i. *n* = 49 interviews ii. *n* = 615,815 calls iii. *n* = 20 interviews iv. *n* = 42,796 non-conveyed incidents	Mixed methods including: i. paramedic, manager, commissioner interviews ii. ambulance dispatch data iii. Qualitative telephone advice data iv. linked ambulance, hospital and mortality data	To understand differences in non-conveyance between ambulance services	Different triage systems in different services; no description of on-scene triage process	Alternative routes of care include referrals to: i. GP out-of-hours service (face to face or via telephone) ii. pharmacy iii. MIU iv. rgent care centre v. social worker vi. psychiatric pathways vii. Community services (home attendance)	– Non-conveyance to ED was facilitated by: i. formal referral pathways ii. informal relationships with local services iii. Organisational facilitation of connectivity between ambulance service and other emergency and urgent care services. – Ambulance trusts with higher rates of non-conveyance: i. had higher skilled paramedics ii. better valued training/skill of these skilled paramedics iii. Better organizational support iv. lower ED rates within 3 days of non-conveyed incident.	**Positive support** – Non-conveyance variation between ambulance services is due to: i. staff skill (e.g. advanced paramedics) ii. perceived value of advanced paramedics iii. Perceived risk adverse views of senior management iv. commissioning of services. – Standardisation of successful processes between ambulance services could reduce unwarranted differences in non-conveyance rates.	4
Pickstone 2019 [33]	UK *n* = 1084	Retrospective audit of referral services	To determine if referral service reduces ED attendances	No description of triage process	– Referral to @home team (which offers 25 acute in-home clinical care pathways)	– 755 (72%) referrals (including ambulance, community services and acute settings) over a 3-month period were accepted, with an estimated 397 ED attendances prevented. – This reduced total number of ED attendances by 0.3%.	**Low support** – The @home referral service reduces ED attendances by a small amount. – Investment of local health services does not have a sufficient impact on service delivery.	4
Schaefer 2002 [34]	USA *n* = 1016 in intervention *n* = 2617 in control	Prospective cohort study compared with a matched retrospective control cohort	To evaluate if EMTs can correctly triage patients alternate care destinations	Two criteria: i. non-urgent severity code ii. one of 24 diagnosis codes	Referral to: i. urgent care centres ii. walk-in clinics iii. GP practices accepting walk-in patients	– Intervention group resulted in: i. increased clinic care (8.0%;4.5%) ii. decreased ED conveyance (44.6%; 51.8%). – Patients reported high satisfaction.	**Positive support** – Alternate care destinations can safely reduce ED visits and provide satisfactory care. – Further investigation of ways to ensure appropriate care of non-urgent patients is needed.	3
Schmidt 2000 [35]	USA *n* = 1300	Prospective cohort study with linked retrospective EMS chart review	To evaluate if emergency medical technicians can safely apply protocols to assign transport options	Series of triage protocols for categories of complaints (e.g. musculoskeletal injuries)	Referral to primary care provider	– There was no difference in classification of transport decision between EMTs and first responders (e.g. fire departments). – 3-11% of patients that were determined not to need ambulance had a critical medical event. – Based on occurrence of critical events, protocol sensitivity was high (95%) and specificity low (33%).	**Low support** – A better triage tool or improved triage adherence is required for EMTs to appropriately triage patients to alternate care routes.	4
Schmidt 2001 [36]	USA n = 1300 (same sample as above)	Prospective cohort study with linked retrospective hospital chart review	To evaluate if emergency medical technicians can safely apply protocols to assign transport options	Series of triage protocols for categories of complaints (e.g. musculoskeletal injuries)	Referral to primary care provider	– 9% (13/140) of patients who were diverted away from ED were under triaged. – Patients with psychiatric complaints and dementia are at higher risk of under triage.	**Mixed support** – Protocols must be created and refined to minimise undertriage rates and ensure correct care pathways for patients.	4
Snooks 2004 [37]	UK *n* = 409 in intervention *n* = 425 in control	Cluster randomised controlled trial and semi-structured interviews	To i. evaluate effectiveness of direct transport of patients to Minor Injury Unit (MIU) ii. describe factors that impact MIU use through interviews with ambulance crews	Protocol outlining 23 minor injuries eligible for transportation to MIU	Transportation to an MIU	– Alternate transportation scheme: i. did not increase non-ED conveyance in intervention group (25.9%; 23.1%) ii. decreased job cycle time, time to treatment and time in unit compared to ED iii. Improved patient’s rating of care. – Ambulance crews reported that location, patient needs, job times, improved service delivery and handover encouraged use of MIU.	**Positive support** – Despite underuse of MIUs, there are no adverse consequences for correct use and many potential benefits.	2
Snooks 2004 [38]	UK *n* = 251 in intervention *n* = 537 in control	Phase 1: Protocol development Phase 2: Prospective cohort with matched control group	To develop and evaluate ‘Treat and Refer’ protocols	Treat & Refer protocols; training delivered to intervention crews (2-day course)	Referral to community based services (GP, district nurse, etc.)	– Referral scheme: i. did not increase non-conveyance in intervention group (37.1%; 36.3%) ii. improved documentation iii. Increased patient satisfaction iv. increased job times v. yielded safety concerns (5.4% of non-conveyed patients were admitted to ED w/i 14 days).	**Mixed support** – Referral scheme did not reduce unnecessary ED conveyance, although patient satisfaction was improved. – There were some concerns with the safety of referral protocols and further research is needed.	3
Tohira 2016 [39]	Australia n = 67,387	Assessment of past patient care records	To evaluate if paramedics can safely identify patients who can be managed in the community	Ordinal triage scale to determine acuity; no clinical guidelines to determine transport	Referral to health services in the community	– 4.8% of ED-transported patients were identified as potentially suitable for community-care. – 53.6% of these were admitted to hospital after direct ED conveyance. – Patients identified as suitable for community care were more likely to require subsequent ambulance request, ED visit and hospitalisation within 24 h than those who were not.	**Low support** – Paramedics were unable to accurately and safely triage patients to non-ED alternatives; this approach is high risk and requires further evaluation.	4
Verma 2018 [40]	Canada *n* = 1851	Retrospective cohort study	To examine associations between paramedic home care referrals and use of services (911 emergency call, ED, home care)	No formal triage criteria	Referral to community services via Community Care Access Centres	- Referrals reduced 911 emergency calls by 10% and ambulance transport to ED by 7%.	**Positive support** – Paramedics can successfully refer patients to community care access centres. – This has promising benefits for reducing future emergency care access including reduced emergency calls and ED conveyances by ambulance	4
Vicente 2014 [41]	Sweden *n* = 410 in intervention *n* = 396 in control	Randomized controlled trial	To evaluate feasibility and safety of alternate transport to geriatric care	Decision support tools for 11 predefined conditions	Transportation to: i. geriatric care iv. community emergency care centre	– 20% of patients were transported to alternate route of care. – 6.7% of non-conveyed patients required transfer to ED w/i 72 h.	**Positive support** – Ambulance nurses can appropriately triage to alternate routes of care. – Such schemes can prevent inappropriate use of the ED and improve care of older adults.	2
Villarreal 2017 [42]	UK *n* = 23,395	Audit of routine ambulance data	Semi-structured paramedic interviews	Triage criteria covers 19 specific incident types	Referral via GP to: i. intermediate care teams ii. social services iii. Community hospitals iv. referral to patient’s own registered GP	– 78% of those who were referred to GP via telephone were not transported to hospital. Patients were more likely to be referred to GP if they were: i. women ii. older – assessed by GP face-to-face.	**Positive support** – Collaboration between paramedics and GP can reduce unnecessary ED transfers. – Recommendation for follow-up of hospital outcome and use of services in subsequent days to assess overall impact and safety of scheme.	4

*Abbreviations*: *ED* Emergency Department, *EMS* Emergency Medical Services, *GP* General Practitioner, *MIU* Minor Injury Unit; *RETTS* Rapid Emergency Triage and Treatment System; *UK* United Kingdom, *USA* United States of America

**Fig. 2:**
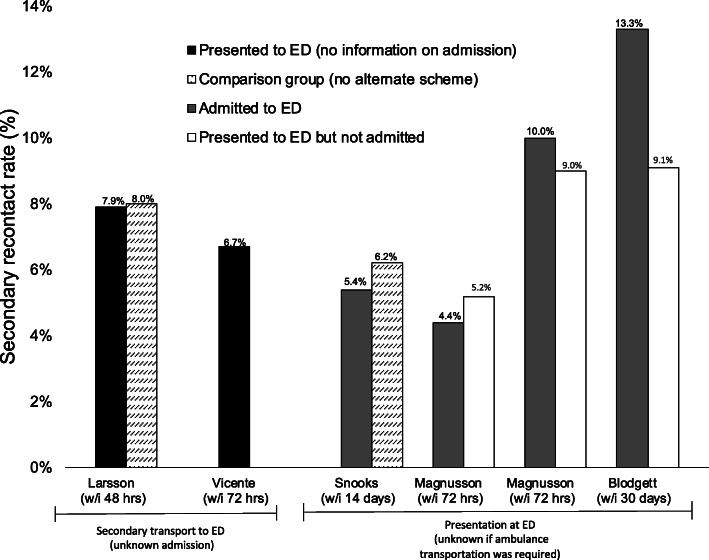
Secondary recontact rates (transport, ED presentation, ED admission) in patients who were referred to an alternate route of care

We identified two main themes: patient safety and impact on operational efficiency. Most studies suggested that paramedics were able to accurately triage patients to the correct pathway of care [31, 32, 35–37, 40, 41]. For example, several studies compared decisions between paramedics, first responders (e.g. fire departments) and senior medical experts reporting high sensitivity (> 94%) and lower specificity (< 58%) [31, 35, 36]. Rates of secondary contact with emergency medical services (e.g. calls, transport, ED presentation) were reported in six studies (Fig. 2). Secondary presentation at ED occurred in 5.4–22.4% of all patients who were referred to an alternate care route (range: 48 h to 30 days) [25, 28–30, 38, 41]; approximately half were subsequently admitted [25, 29, 30]. Only two studies compared rates in intervention and control group, reporting no difference in secondary ED presentation (7.9% vs 8.0%) [28] or admission rates (5.4% vs 6.2%) [38]. Not all studies reported that paramedics could correctly triage patients. For example, one study reported that 53.6% of patients identified by paramedics as eligible for alternative routes of care were subsequently admitted to hospital and were also more likely to experience adverse events than those who conveyed directly to ED [39]. Other studies highlighted concerns of under triage as shown by high secondary recontact rates, overturned decisions by medical experts (19.5–22%) [31, 42] and occurrence of critical events in those incorrectly triaged away from ED (3–11%) [35, 36]. Psychiatric presentations and patients living with dementia were considered at particular risk of under triage [36].

Studies reported that alternative care routes improved operational efficiency by decreasing ambulance job cycle times [28, 29, 37], decreasing ED conveyance rates [26, 28, 32, 34, 40, 41], improving patient documentation [38], increasing clinic care destinations [34], decreasing hospital admissions [25, 28, 33] and improving or maintaining patient satisfaction [31, 34, 37, 38]. Nevertheless, there were conflicting findings that reported longer job cycle times [38] or showed that alternative care schemes had no impact on decreasing ED conveyance rates [37, 38].

### Qualitative, evidence based studies

Characteristics of the 12 qualitative studies are provided in Table 2. Study characteristics of the qualitative studies were similar to above, with a third of studies (*n* = 4) in each of the UK and Sweden, two studies in Ireland and one in each of Canada and the USA. As in the quantitative studies, the main alternate care routes comprised of primary care referrals, referral or transport to urgent or community care centres and transport to minor injury units. Data was primarily collected from interviews with paramedics (*n* = 6) or ambulance nurses (*n* = 2), patients (*n* = 3), emergency medical physicians (n = 2) or senior managers/commissioners of ambulance trusts (n = 2). There was unanimous support from all studies that schemes providing alternate routes of care than direct ED-conveyance can deliver vast benefits, although several barriers were recurrently identified.

**Table 2:** Summary of qualitative, evidence-based studies (*n* = 12)

First author year (ref)	Country & sample size	Data collection	Aim	Description of alternate route of care	Findings and concluding evidence (and level of support)	Level of Evidence ^**+**^
Blodgett 2017 [13]	UK *n* = 8	Semi-structured paramedic interviews and observation	To investigate paramedic’s perspectives on barriers and motivations on GP referrals.	Referral to GP via ambulance scheme	Paramedics described: i. time, process and training as the major barriers ii. their open mindedness and positivity about utilising the scheme iii. Frustrations with GP decision making iv. gaps in awareness and understanding of scheme.	6
Brydges 2015 [43]	Canada *n* = 23	Semi-structured paramedic interviews	To investigate paramedic’s perspectives on challenges and opportunities with referrals	Referral to community services via Community Care Access Centres	Paramedics reported: i. confusion in their role ii. inadequate knowledge on referral iii. no feedback on success of referral iv. lack of accountability on use of scheme v. desire to provide best care for patient.	6
Bury 2005 [44]	Ireland *n* = 11 (surveys) *n* = 5 (interviews)	Questionnaire surveys from GP cooperatives Semi-structured interviews with senior management/ GPs	To describe the preparedness and contribution of GP co-operatives to manage emergencies in the community	Referral to GP co-operatives providing out-of-hours services	– 3/11 GP co-operatives had formal liaisons with ambulance service. – 4/8 GP co-operatives received referrals from ambulance services (3 unknown). – GPs reported uncertainty and anxiety in dealing with 999 referrals due to lack of established structure compared to normal practice.	6
Hoglund 2019 [45]	Sweden *n* = 20	Semi-structured interview with ambulance nurses	To explore ambulance nurses’ experiences of non- conveying patients to alternate levels of care	Transportation or referral to primary healthcare or other healthcare facility (optional consultation with GP)	Nurses reported: i. desire to find the best pathway of care ii. that non-conveyance is demanding and complex task and the main challenges were: • misconceptions by patients about ambulance need • resources shortages iii. Lack of training and mandates to convey to appropriate level of care.	6
Jones 2005 [46]	USA *n* = 1058	Cross-sectional surveys with ED patients	To assess if patients were willing to accept non-conveyance alternatives including different destination and/or modes of transport	Transport to urgent care centres or primary care physician offices or referral to telemedicine	Patients were: i. willing to consider transport to non-ED alternatives (69%) ii. more likely to consider alternatives if they were: younger, non-white race, lower patient acuity and had lower self-perceived illness severity.	6
Knowles 2018 [47]	UK *n* = 49	Semi-structured interviews with managers, paramedics and lead healthcare commissioner from 10 ambulance services in England	To explore variation in how ambulance services address non-conveyance for calls ending in telephone advice and discharge at scene	Transport or referral to range of different facilities: i. walk-in centre ii. MIU iii. GP	Differences between regional ambulance trusts had a substantial effect on use of alternative options. Main differences included: i. senior management’s approach to non-conveyance options (e.g. opportunity vs risky endeavour) ii. paramedic skill and training to appropriately triage patients to alternative care routes iii. Availability of services and care pathways that facilitate non-conveyance.	6
Lederman 2019 [48]	Sweden *n* = 11	Semi-structured interviews with ambulance nurse	To explore ambulance clinician’s experiences of assessing non-conveyed patients	Alternate transport or referral to: i. primary healthcare unit ii. MIU iii. Community care practitioner	Ambulance nurses reported: i. high willingness and recognition of benefits of non-conveyance alternatives ii. lack of confidence in decision making iii. Lack of organisational support for decision-making iv. insufficient training and feedback on non-conveyance decisions (e.g. missed learning opportunities).	6
Miles 2019 [49]	UK *n* = 143	Surveys with paramedic using quantitative and qualitative assessment of 6 patient vignettes	To: i. examine if paramedics can accurately identify the most clinically necessary destination ii. .understand what contributes to decision making.	Alternate: i. transport to MIU ii. referral to GP iii. Referral to pharmacist	– Paramedics decisions were made with 69% accuracy. – Sensitivity of correctly choosing ED: 0.90. – Specificity of correctly choosing non-ED routes: 0.49. – Decision-making was influenced by: i. patient safety ii. risk aversion (e.g. fear of litigation/consequences) iii. Comparison of patient’s presentation to normal condition.	6
Power 2019 [50]	Ireland *n* = 375	Survey of stakeholder opinions including: i. emergency medicine consultants ii. paramedics iii. Advanced paramedics	To understand stakeholder views on implementing a Treat and Referral care pathway to minimise ED attendance	Alternative routes not described, but cover all situations where an ambulance crew offers a disposition other than ambulance transport to an ED	– Stakeholders expressed clear support to introduce program into ambulance service. – There was a consensus that program would improve patient care and clinical judgement of practitioners. – The following suggestions were made: i. clinical audit to demonstrate improved care ii. initially implement program for advanced paramedics iii. Safety and efficacy of different clinical conditions must be evidence-based before implementation across trust.	6
Rantala 2018 [51]	Sweden *n* = 111	Cross-sectional surveys with patients assessed as non-urgent (yellow or green by RETTS)	To explore patient’s experiences of the person-centred climate (and construct validity of person-centeredness dimension)	Referrals to other level of care (e.g. primary care, GP visit at home)	Patients reported that: i. the environment was highly person-centred ii. their clinical complaints were taken seriously.	6
Snooks 2005 [52]	UK *n* = 15	Three focus groups with ambulance crews: 1x pre-intervention 1x post-intervention 1x control group.	The authors describe ambulance crew’s views about non-conveyance to hospital including decision making process, alternate route or care and use of triage protocols	Referral to community based services (GP, district nurse, etc.) using Treat & Refer protocols as described in [38]	Paramedics described: i. positivity about implementing referral scheme across the ambulance service ii. difficulties with the scheme including: •more training for paramedics •patients who were unreceptive to referral iii. Ensuring wider support of primary care and community services.	6
Vicente 2013 [53]	Sweden *n* = 11	Semi-structured interviews with older patients who were referred	To describe the patient experience of being offered an alternative care pathway to ED conveyance	Transportation to geriatric care or community emergency care centre as described in [41]	Patients reported: i. a preference for an alternative to direct conveyance to ED ii. a desire to be involved in the decision making.	6

*Abbreviations*: *ED* Emergency Department, *EMS* Emergency Medical Services, *GP* General Practitioner, *MIU* Minor Injury Unit; *RETTS* Rapid Emergency Triage and Treatment System, *UK* United Kingdom, *USA* United States of America

The main barriers identified by paramedics to successful implementation were training, organisational support and process. First, the absence of training and knowledge on referral triage and processes [13, 43, 45, 47, 48, 52] often manifested as a lack of confidence in both themselves and the system [13, 48], with some paramedics expressing concerns about being held accountable or facing legal action for under triaging patients [47, 49]. Next, there were concerns that the absence of a mandate from their organisation led to a lack of responsibility to refer and, thus, underuse of the scheme [43, 45]. Organisational support from ambulance services and partnerships with primary or community care pathways were identified as crucial components of an effective scheme [45, 47, 48, 52]. Paramedics did not receive feedback on patient outcomes from their own organisation or the referral destination; they reflected that this was demotivating and a lost learning opportunity [43, 48]. Finally, although uncommon, paramedics expressed minor frustrations about the referral process itself [13], perceived time spent to refer [13, 43] and misconceptions from patients who wanted direct conveyance to ED [45, 52].

Consistent with views from paramedics, interviews with stakeholders identified the importance of established structures between organisations [44, 47] and sufficient training and knowledge [50] of paramedics. Power et al. [50] was the sole paper to contrast viewpoints between emergency medical consultants and paramedic towards “treat and referral” schemes. Emergency medical consultants more commonly reported that referral/transport to ED alternatives should be limited to advanced paramedics (57.2% vs 22.6%; *p* < 0.001), that paramedics should inform the patient’s GP after each referral/alternate transport (88.9% vs 47.5%) and were less optimistic that the scheme would improve ambulance availability (55.6% vs 83.9%) [50]. Finally, patients expressed both a willingness [46] and a preference [53] to be directed to an alternate scheme rather than conveyed directly to ED. They emphasised their desire to contribute to the decision-making process [53], and felt that their views were taken seriously [51, 53].

### Consensus- based studies

Six of the 10 consensus-based articles were based on the American EMS systems, three concerning UK systems and one on Australian systems. These articles were primarily opinion-based (*n* = 2 editorials, n = 2 viewpoints, *n* = 3 commentaries), but also included two policy statements, a scheme overview and one promising RCT study protocol. Details of all studies are provided in Table 3. Note that results from the study protocol are not publishable due to “unresolvable inconsistencies in data” [57]. The opinion-based pieces and the policy statements both highlighted the promising potential for such schemes, with particular emphasis on projected cost savings [54, 58, 62, 63]. A policy statement, written in 2001 by the Emergency Medical Services Committee and reaffirmed in 2008 by the American College of Emergency Physicians, highlighted seven key elements that should be considered when implementing alternative routes of care [59, 60] (see Table 3). Consistent with above, patient safety and accuracy of paramedic triage decisions were raised as main areas of concern [16, 58, 63, 64]. Sawyer et al. [64] was the sole article to recommend against implementation of such schemes citing concerns of insufficient supporting evidence, under triage having an adverse effect on patient safety and vulnerable patients being disproportionally affected.

**Table 3:** Summary of consensus-based articles (*n* = 10)

First author year [ref]	Article type	Country	Description of alternate route of care	Article description and author recommendations	Level of Evidence ^**+**^
Alpert 2005 [54]	Commentary and economic cost analysis	USA	Transport to a physician’s office or health centre	– Between 12 and 16% of Medicare covered transport to ED were avoidable. – Federal government could save $283–560 million+ per year if EMS ambulances can refer to non-ED alternatives.	7
Altoft 2003 [55]	Scheme overview	UK	Intermediate care scheme that provides nursing care, physiotherapy, occupational therapy and rehabilitation	– Referrals to scheme from ambulance crews are rare. – Paramedics who have used the scheme have positive reports. – Increased use of scheme can prevent hospital conveyance and admission and provide better patient care.	7
Arendts 2011 [56]	Study protocol ***Note****: study results were not published due to “unresolvable inconsistencies in data”* [57]	Australia	Referral to a rapid (w/i 4 h) response primary care service in the patient’s own residence	– Protocol outlines: 1. randomisation to: i. intervention (rapid response service) ii. control (direct ED conveyance) 2. assessed outcomes will be: i. unplanned medical attention w/i first 48 h ii. clinical hospital outcomes iii. Cost benefit analysis.	7
Asplin 2001 [58]	Editorial	UK	To discuss how and who should identify patients that can be triaged safely away from ED and how to reduce unnecessary ED visits	– Several key issues are highlighted: i. paramedic’s ability to triage and make decisions ii. patient safety of non-conveyance alternatives iii. Cost effectiveness of non-conveyance alternatives iv. access barriers experienced by EMS staff and patients.	7
Blodgett 2017 [16]	Viewpoint	UK	To discuss an ambulance trust’s GP referral policy as an alternate to direct conveyance	– Overview of a collaborative telephone referral policy between on-scene paramedic and GP is provided. – Early evidence suggests that 61% of patients referred to GP do not attend ED within 30 days. – There are some positive results, but critical appraisal of patient safety and re-contact rates is necessary.	7
Emergency Medical Services Committee 2001 [59]	Policy statement	USA	No specific alternate route of care described	The American College of Emergency Physicians and the National Association of EMS Physicians identify the need for alternative routes of care and outline key elements that should be included: i. physician medical director oversight ii. assurance of patient safety in development/intervention iii. Training for ambulance personnel iv. compliance with dispatch criteria v. no circumvention of 999/911 system vi. consistent with medical necessity vii. Appropriate compensation for EMS systems.	7
American College of Emergency Physicians 2008 [60]				The above policy was reaffirmed in 2008.	7
Hsiao 1994 [61]	Commentary	USA	To propose a regional community health monitoring and referral system	Authors overview a model in which a centralized monitoring agency could coordinate EMS use and link patients to required levels of care, support, education and interventions.	7
Morganti 2014 [62]	Commentary	USA	To propose changes in payment policy that allow and promote alternatives to direct ED conveyance	– Current American payment policies discourage non-conveyance to ED. – There are theoretical benefits of alternate transport settings and on-scene treatment alternatives. – Assessment of alternate pathways of care is a high priority.	7
Munjal 2019 [63]	Viewpoint	USA	To discuss barriers and consequences of alternative payment model that allows EMS agencies to be reimbursed for non-conveyance to ED	– Alternate care routes include: i. nurse triage ii. treatment by health care practitioner on scene or via telephone iii. Transportation to urgent care centre or primary care physician. – Main barriers are: i. patient safety ii. quality measurement and assurance iii. Feasibility of payment models in different jurisdictions. – Emphasised that the alterative model is a major advancement for out of hospital care.	7
Sawyer 2017 [64]	Editorial	USA	To highlight concerns of alternatives to ED conveyance (including transport to primary care, general medical clinics, urgent care centres, and other social or psychological services)	– Several concerns about implementing alternative transport options: i. limited evidence to support ‘theoretical’ claims of benefit to ED use, cost saving and enhanced primary care access ii. patient safety as a result of under triage by paramedic iii. Alternative destinations will disproportionately affect critically ill and vulnerable patient populations.	7

*Abbreviations*: *ED* Emergency Department, *EMS* Emergency Medical Services, *GP* General Practitioner, *MIU* Minor Injury Unit, *RETTS* Rapid Emergency Triage and Treatment System; *UK* United Kingdom, *USA* United States of America

## Discussion

This broad scoping review provided an overview of 41 articles examining alternate routes of care to direct ED conveyance. Despite heterogeneous study characteristics and diverse alternative care pathways, there was strong consensus from patients, paramedics and other healthcare practitioners of the benefits of alternative care schemes. Even so, several key barriers were emphasised. Positive support broadly covered three topics. First, non-ED alternatives were reported to improve operational efficiency by decreasing ED conveyance, reducing incident time and providing projected savings across the emergency health care sector. Next, there was clear recognition by paramedics, stakeholders and patients that alternate care schemes can provide optimal patient care pathways. Finally, despite unanimous recognition of the importance of patient safety when using or implementing these schemes, there was mixed evidence as to whether paramedics could accurately and safely triage patients to the appropriate level of care. Other barriers included insufficient evidence of patient safety, unsatisfactory training, and a lack of formal partnerships between ambulance services and supporting services.

### Recommendations for a successful alternate route of care

Analysis of patient data and interviews with those involved in the care pathway (e.g. patient, ambulance clinician, stakeholder) suggested that successful schemes share four key features. First, clear triage tools are crucial in guiding accurate decision making of ambulance clinicians. Formal triage pathways can ensure that patients are referred or conveyed to the appropriate destination. For example, tools such as the UK-based Paramedic Pathfinder [31] or the Swedish-based Rapid Emergency Triage and Treatment System (RETTS) [28–30] allow triage outcomes that direct patients to a specific alternative care route (e.g. GP referral, community care centre). Without a guiding framework, non-specific triage tools can lead to incorrect triage decisions [34, 39]. There is no universally accepted triage tool that could be applied everywhere as the availability of schemes differs by health care system, both within and across country. Additionally, geographical considerations play an important role as densely populated urban areas may be able to facilitate non-conveyance of patients better than rural areas with fewer resources. As such, formal triage tools must be specific to individual healthcare systems and provide clear support to guide the decision making of ambulance clinicians.

Second, additional training on correct use of alternative care schemes including triage tools, overview of processes and learned examples is necessary. Although higher skilled ambulance clinicians (e.g. advanced paramedics, ambulance nurses) may have improved decision-making processes, there was a persistent belief that paramedics of all skill levels should still be trained to appropriately use such schemes. Inadequate training likely explains why paramedics have perceived low confidence and hesitation to use these schemes [43, 48, 52].

Third, formal liaisons and partnerships between ambulance services, primary care, urgent care centres, minor injury units or psychiatric and social teams are crucial in facilitating referral or alternate transportation of patients. Without well-established pathways of care, ambulance clinicians are forced to rely on ad hoc decisions and, as a result, are often unsuccessful in finding an appropriate alternative source of care [32]. This must be a consideration when considering implementation of schemes in different countries or regionalised ambulance services, which may limit availability of alternate facilities. Finally, the most important and persistent recommendation in quantitative, qualitative and consensus-based studies was the need for adequate evidence demonstrating patient safety. It was commonly suggested that the current evidence was not sufficient to justify implementation of such schemes; this is described further below.

### Ongoing concerns and challenges

Research in this area is increasing, contributing to and reflecting positive developments in the paramedicine profession [65, 66]. Half of the studies were published within the last 5 years, with only one study coming before the turn of the century [61]. Expert opinions and viewpoints identify the importance of providing alternatives to direct ED conveyance, while qualitative studies are fundamental in describing the views of ambulance clinicians to understand how human factors can ensure optimal use of these schemes [67]. However, the quality of existing evidence is poor, particularly as it pertains to the most important outcome of patient safety.

The overall level of evidence is low, with only two studies using an RCT design (Level 2) [37, 41]. RCTs are essential to assess the impact of an intervention on patient outcomes [68]. Here, they enable alternative care schemes to be compared to ambulance services without alternative options and may provide a holistic overview of patient outcome differences. Conversely, nearly all relevant studies failed to formally assess whether the schemes are safe and instead, considered accuracy of conveyance decisions (e.g. between ambulance clinicians and expert medical consultants) [31, 35, 36, 49] or rates of secondary contact [25, 28–30, 38, 39, 41] as proxies for patient safety. While accuracy of decision making may inform future outcomes, it is important to consider both short and long-term patient safety. Secondary contact rates may not provide a reliable indication of individual outcomes. For example, patients may contact emergency services for a reason unrelated to the original incident or recontact rates may be inaccurate if data from all possible health services (e.g. GP, ambulance, ED, etc.) are not obtained. Additional secondary contact with emergency services is common regardless of patient destination; only one study considered how recontact rates in a clear intervention group (e.g. with non-ED conveyance alternatives) compared to a control group, reporting there was no difference in secondary transport within 48 h (7.9% vs 8.0%) [28]. Failure of studies to appropriately consider the impact on the whole system has previously been highlighted [49].

It can be challenging to generalise findings across countries or between services in the same country, as the structure of the EMS system poses unique challenges. For example, there may be higher potential for alternative schemes in the publicly funded UK system which may allow better linkage within healthcare facilities, while privately funded and delivered health care systems, such as the USA, may encounter different challenges [19, 69, 70]. Similarly, Swedish ambulances are staffed with qualified nurses, which may provide them with a larger scope of practice in their decision making than emergency medical technicians in other countries [71, 72]. Most articles proposing cost efficiency of these schemes are US-based and consider specific regional EMS organisations and privatized primary care; this makes it difficult to draw conclusions for a federally funded healthcare system.

Finally, it is important to identify an acceptable under triage rate in non-ED conveyance situations [10]. High sensitivity of tools suggest that ambulance clinicians are able to accurately assess who needs to go to ED, while the lower specificity indicates that they are less able to identify who may benefit from an alternative care route [31, 35, 49]. Over conveyance to ED is preferable to a model that regularly under triages individuals to lower levels of care than is required, and as such, triage tools for non-ED alternatives may necessitate a high-sensitivity, low specificity approach [10]. Further evaluation and assessment of the safest level of under triage requires further investigation.

### Limitations

Due to the heterogeneity of the literature, the identification of relevant articles was challenging; despite a wide and inclusive search strategy, it is possible that relevant articles were not identified. Furthermore, due to ongoing innovation of the paramedicine field, we believe that there is a significant amount of discourse on alternate routes of care that has not been formally researched or published. It is important to disseminate results that have undergone formal peer-review to assure the highest quality of evidence and to help establish the evidence base. Many ambulance services work in isolation as they navigate and identify these alternative care pathways; communication between services both within and between countries is crucial for a collaborative effort to confront these issues. Details of triage tools and non-ED conveyance routes of care are insufficiently provided in study articles. Newton et al. [31] provide a detailed overview of referral routes, the accompanying triage tool and the accuracy of decision making in one UK ambulance trust; future studies should provide similar overviews, whilst expanding to assessment of patient outcomes.

As we aimed to identify non-ED conveyance alternatives that could be adapted to a wide range of intermediate patients, we excluded studies that provided specific clinical pathways; for example, community paramedicine pathways that provide proactive home visits, specific fall pathways or alcohol detoxification centres [73–75]. However, synthesis of the effectiveness of these targeted schemes is needed. A combination of specific clinical alternatives along with general pathways for intermediate patients can help ensure the highest number of patients can be referred to the optimal level of care.

Finally, the variability in study design, scheme, protocol, outcome and sample population rendered it difficult to synthesize and summarize the evidence. While several studies reported similar outcomes including decision accuracy matrices and recontact rates, heterogeneity of these outcomes made us unable to consider a formal meta-analysis. Additionally, several studies grouped patients who were not immediately conveyed to ED together; thus, it was not possible to assess if referrals or transfers to alternate care routes provide a better safety net than self-care at home.

### Future steps

Given the widespread use of alternative schemes in the UK and Sweden, and increasing implementation in North American countries, it is crucial to commission large scale studies evaluate patient outcomes for those conveyed directly to ED, those left at home and those referred to alternate routes of care. Notwithstanding the impact of decreasing emergency department burden and cost effectiveness, patient safety must remain the most important outcome. Studies consider the entire patient journey, which may involve linkage of data from several emergency or primary care services. There must be improved collaboration between ambulance services within a single country and shared opportunities to learn from other countries. Given that schemes are linked to paramedic skill, training and education (e.g. research nurse, degree paramedic, emergency medical technician), the growing positive advancements in paramedic education in recent years may provide new opportunities and additional scope for these new patient pathways [65, 66].

## Conclusions

This scoping review provided a broad summary of current evidence and consensus-based articles that examined alternate routes of care for the intermediate, non-urgent patients. Most evidence suggests that such schemes can improve operational efficiency, reduce ED conveyance and provide an optimal care pathway for the patient. Paramedics, GPs, patients and stakeholders all expressed a high willingness and recognised the benefits of such a scheme. Still, the majority of the studies lacked methodologically rigorous design and evidence of safe outcomes; there remains a significant need to examine patient safety in non-ED conveyance schemes.

## Data Availability

Data sharing is not applicable to this article as no datasets were generated or analysed during the current study.

## Appendix

### Search strategy in PubMed

1. paramedic*
2. ambulance*
3. “emergency service”*
4. “emergency medical service”*
5. “emergency technician”
6. “emergency practitioner”
7. “emergency dispatch”
8. “first responder”
9. “emergency rescue”
10. “emergency triage”
11. “emergency care practitioner”
12. 1 OR 2 OR 3 OR 4 OR 5 OR 6 OR 7 OR 8 OR 9 OR 10 OR 11
13. referral*
14. triage*
15. “emergency department”
16. “accident and emergency”
17. “A&E”
18. “non convey”*
19. non-convey*
20. deflect*
21. 13 OR 14 OR 15 OR 16 OR 17 OR 18 OR 19 OR 10
22. 12 AND 21

## Abbreviations

ED: Emergency department
EMS: Emergency medical services
GP: General practitioner
PRISMA-ScR: Preferred Reporting Items for Systematic Reviews and Meta-Analyses extension for Scoping Reviews
RETTS: Rapid Emergency Triage and Treatment System
RCT: Randomised control trial
UK: United Kingdom
USA: United States of America
